# Aggressive Differentiated Thyroid Cancer due to EML4e13-ALKe20 Fusion: A Case Presentation and Review of the Literature

**DOI:** 10.1155/2021/8837399

**Published:** 2021-02-15

**Authors:** Rodhan Khthir, Zainab Shaheen, Prasanna Santhanam, Saroj Sigdel

**Affiliations:** ^1^Marshall University, School of Medicine, Huntington, WV, USA; ^2^Johns Hopkins University, School of Medicine, Baltimore, MD, USA

## Abstract

**Background:**

Differentiated thyroid cancer (DTC) is an indolent malignancy. It rarely presents with aggressive local invasion and/or distant metastatic disease. *Patient findings*. We describe a case of a 30-year-old man with a locally aggressive form of papillary thyroid cancer with *EML4e13-ALKe20* fusion (*EML4*: echinoderm microtubule-associated protein-like 4; *ALK*: anaplastic lymphoma kinase). He presented with right-side cervical lymphadenopathy with a highly suspicious right-side thyroid nodule. Total thyroidectomy and level IV lymph node resection showed extensive bilateral disease, with extrathyroidal and extranodal extension. FDG-PET CT scan following surgery confirmed the presence of significant residual disease in the neck area. He underwent bilateral lateral lymph node dissection followed by radioactive iodine treatment. Somatic mutation testing showed *EML4e13-ALKe20* fusion. *Summary*. This case represents an aggressive form of DTC with *EML4e13-ALKe20* fusion. The rapid progression of clinical signs and symptoms and the local extension beyond the thyroid and lymph nodes with the persistence of high-volume local disease after thyroidectomy highlight the aggressive nature of this mutation and the importance of performing genetic analysis to guide future treatments and determine prognosis.

**Conclusion:**

This case highlights the importance of using molecular diagnostics in patient care, especially if the presentation is unusual for DTC. A thorough evaluation of the tumor pathology and the somatic mutational profile analysis are important for obtaining vital therapeutic and prognostic guidance.

## 1. Introduction

Over the last several years, significant progress has been made in understanding the genetic mechanisms behind thyroid cancer and creating molecular tests for diagnosing cancer in thyroid nodules.

*ALK*-associated fusions and point mutations are found in various tumors, including anaplastic large cell lymphoma, non-small-cell cancers (NSCLCs), inflammatory myofibroblastic tumor, diffuse large B-cell lymphoma, esophageal squamous cell carcinoma, renal medullary carcinoma, renal cell carcinoma, breast cancer, colon cancer, and neuroblastoma [1], and serve as diagnostic markers and therapeutic targets. Their role in thyroid cancer is less well established.

We present a case of DTC with *EML4e13-ALKe20* fusion with atypical presentation. We conducted a literature review on this rare mutation to better understand its clinical characteristics and potential treatment options.

## 2. Case Presentation

**Figure 1:**
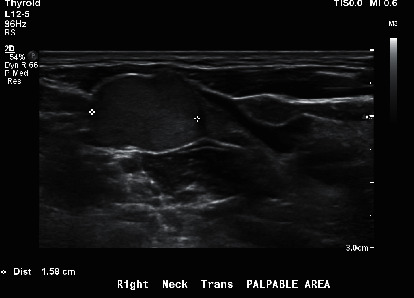
Ultrasound transverse image of the right palpable swelling showing abnormal cervical lymph node with localized invasion.

**Figure 2:**
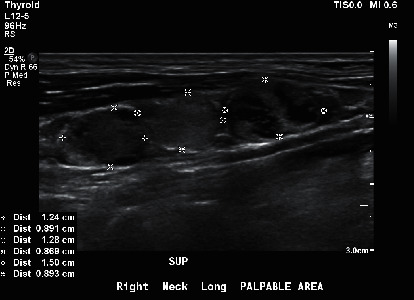
Ultrasound transverse image of lymphadenopathy in the right cervical area with distortion of normal morphology and cystic necrosis.

A 30-year-old male with no significant past medical history presented to his primary care physician in early 2019, with a painless right neck swelling in addition to multiple palpable masses in the area. A neck ultrasound showed multiple irregular and enlarged lymph nodes on the right side of the neck (Figures 1 and 2) along with a calcified right thyroid nodule measuring 17 × 20 × 13 mm. The left lobe of the thyroid was normal. The TSH value was 1.04 mIU/ml, confirming a euthyroid status. The patient reported no history of ionizing radiation exposure and no family history of thyroid cancer or other familial cancer syndromes.

Fine-needle aspiration (FNA) from the palpable lymph node revealed rare atypical epithelial cells and histocytes. Fine-needle aspiration from the right thyroid nodule was performed at an outside facility before referral, and the results were benign (presumably Bethesda class 2). We suspected a sampling error.

Because of the high index of suspicion for thyroid cancer, he underwent total thyroidectomy with central neck dissection and right lateral lymph node dissection at the end of July 2019.

Intraoperative findings showed the presence of extensively involved cervical lymphadenopathy at multiple levels (IV–VI), mainly on the right side. The right cervical level IV and V lymph nodes were tightly adherent to the internal jugular vein and the other large blood vessels. Abnormal paratracheal lymph nodes were dissected from both sides. Postoperatively, the patient developed right recurrent laryngeal nerve paralysis as there was local invasion around the nerve. However, there was no evidence of postsurgical hypocalcemia.

**Figure 3:**
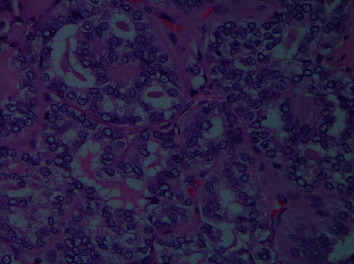
Microscopic examination showing infiltrative tumor with follicular architecture and eosinophilic scalloped colloid. There is overlapping and crowding of tumor cells with cytologic atypia. Numerous pseudoinclusions, nuclear grooves, and ground glass nuclei are also noted.

**Figure 4:**
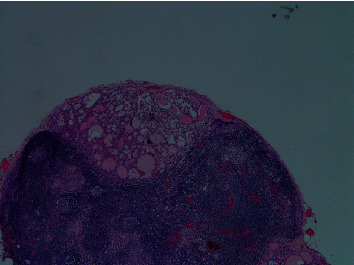
Positive lymph node: the image shows lymph node partially replaced by tumor cells.

The histopathology of the resected thyroidectomy specimen showed multifocal follicular variant papillary thyroid cancer (FV-PTC) infiltrating both lobes and the isthmus with angioinvasion, lymphatic invasion, and microscopic extrathyroidal extension (Figure 3). The right superior and left superior lobe margins were also positive for the tumor. The largest tumor sizes were 3.5 and 1.5 cm in the most significant dimension, and the smallest tumor size was 0.1 cm. The Delphian lymph node and 4/4 right level VI lymph nodes had tumor involvement. Left paratracheal nodes (4/4) also had tumor involvement. A total of 5/9 lymph nodes from the right lymph node dissection at levels IV and V had metastatic tumor involvement. The largest metastatic tumor size was 4.0 cm, and this tumor showed an extranodal extension (Figure 4).

On the initial visit to the endocrine clinic four weeks after surgery, a physical exam was remarkable only to develop hoarseness of voice. No palpable lymphadenopathy was noted. The patient was not on thyroid replacement therapy. The thyroglobulin level was 294 ng/ml (TSH 52.9 mIU/ml). Antithyroglobulin antibodies were undetectable.

**Figure 5:**
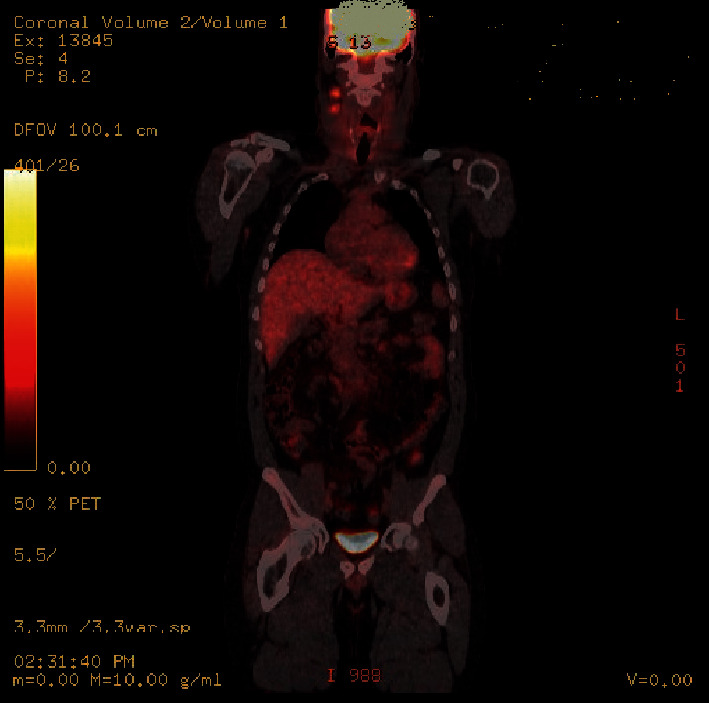
FDG-PET scan showing increase in uptake in two upper cervical lymph nodes.

Because of the patient's DTC's aggressive nature, next-generation sequencing was performed on the tumor tissue (OmniSeq; Integrated Oncology Lab) using multiplexed PCR-based DNA and RNA sequencing. The results showed *EML4e13-ALKe20* fusion, a relatively common fusion described in non-small-cell lung cancer, which creates a more tumorigenic *ALK* kinase domain receptor by partnering with echinoderm microtubule-associated protein-like 4 (*EML4*) [1]. No other molecular alteration was found. Because of the high thyroglobulin level (294 ng/ml; TSH 52.9 mIU/ml), which was suggestive of persistent structural disease or distant metastasis, an FDG-PET/CT scan was performed. It showed two hypermetabolic lymph nodes in the upper cervical chain posterior to the internal carotid artery (SUV of 4.9 and 4.4). There was another hypermetabolic lymph node in the left supraclavicular area (SUV of 3.9). There was no significant uptake in the thyroid bed and no evidence of distant metastasis (Figure 5).

The patient underwent a second surgery (bilateral neck dissection for residual disease), and as per the surgeon's description, there was no macroscopic disease left in the explored area. Pathology showed metastatic papillary thyroid cancer in 2 out of 12 right level II lymph nodes and 6 out of 13 left level III lymph nodes. The size of the largest metastatic tumor was 1.3 cm on both sides. There was evidence of extranodal extension on both sides.

The thyroglobulin level was reduced to 28 ng/ml 10 weeks after the second neck dissection (TSH 83.70 mIU/ml). The patient received 150 mCi I-131 therapy for the treatment of remnant tissue and residual disease.

**Figure 6:**
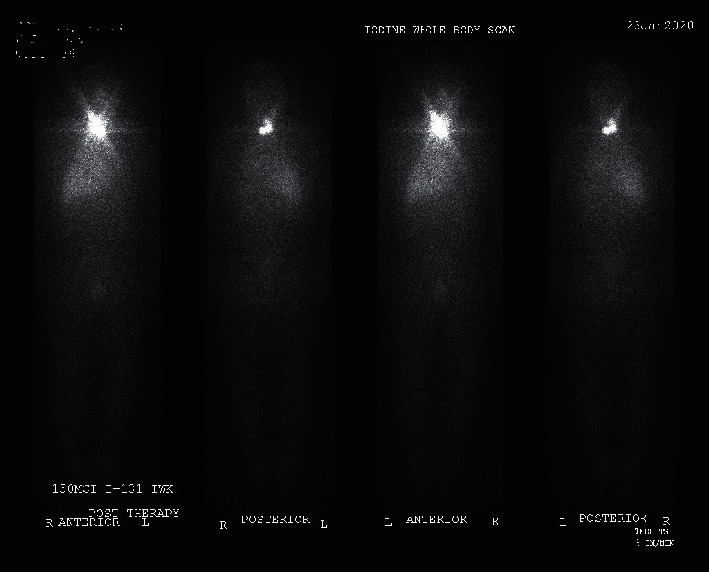
Whole body scan, one week posttreatment with 150 mCi I-131. It shows localized uptake in the neck area with no evidence of distant metastasis.

The posttreatment scan showed localized uptake in the neck area (Figure 6).

Four months after posttreatment with radioactive iodine, the thyroglobulin level decreased to 0.19 ng/ml with undetectable antithyroglobulin antibodies (TSH 0.261 mIU/ml). There was no evidence of local recurrence by physical examination.

## 3. Discussion

The above case represents a highly aggressive form of DTC with an unusual somatic mutation.

Classical molecular abnormalities involving the *MAPK* (mitogen-activated protein kinase) and *PI3K* (phosphoinositide 3-kinase) pathways that play a crucial role in follicular-derived thyroid cancer have been previously described [2]. Point mutations in the gene encoding *BRAF* are the most common genetic alterations in papillary thyroid cancer [3, 4]. Such mutations are found in 40–60% of classic papillary thyroid cancers with or without tall cell features, 20–40% of poorly differentiated thyroid cancers (PDTCs), and 30–40% of anaplastic thyroid cancers (ATCs). Point mutations in the gene encoding *RAS* are found in 40–50% of follicular thyroid cancers and 20–40% of PDTCs and ATCs [5].

Papillary thyroid cancers are also associated with gene fusion and rearrangements involving receptor tyrosine kinases (RTKs), such as ALK, RET, MET, NTRK, FGFR, and ROS1. The activation of most RTK requires molecular dimerization. Rearrangements of RTK-encoding genes lead to ligand-independent dimerization, which results in the constitutive tyrosine kinase activity and signal transduction to the RAS/RAF/MAPK, PI3K/AKT/mTOR, and JAK-STAT pathways [6]. The chimeric genes resulting from RET rearrangements are referred to as RET/PTC and those resulting from NTRK1 rearrangements as RTK. These gene fusions are prevalent in up to 40% of sporadic papillary thyroid cancers [7].

Our patient has an aggressive DTC with a less common gene fusion involving the *ALK* gene (*EML4e13-ALKe20* fusion). ALK fusion proteins have been shown to activate various signaling pathways, among which are the phosphtidylinositol 3-kinase (PI3K)/Akt pathway and the Ras ⟶ Raf ⟶ MEK ⟶ ERK (MAP kinase) pathway with multiple interaction points to mediate the ALK signaling. Such fusion promotes ligand-independent signaling and increases growth and cell invasion [1].

EML4 is a member of the echinoderm microtubule-associated protein-like family. The gene encodes a WD-repeat-containing protein that may be involved in microtubule formation. An abnormal fusion of parts of this gene with portions of the anaplastic lymphoma receptor tyrosine kinase gene generates EML4-ALK fusion transcripts.

The *ALK* gene encodes a receptor tyrosine kinase that belongs to the insulin receptor superfamily (https://www.genecards.org/Search/Keyword?queryString=ALK%20EML). This protein comprises an extracellular domain, a hydrophobic stretch corresponding to the single-pass transmembrane region, and an intracellular kinase domain. It appears to play an important role in the development of the brain and exerts its effects on specific neurons in the nervous system. Chromosomal rearrangements are the most common genetic alterations in this gene and result in the creation of multiple fusion genes which act as oncogenes, leading to constitutive autophosphorylation and activation of ALK tyrosine kinase. For reasons that are not currently understood, it seems that ALK locus is prone for translocation. Nearly 30 different *ALK* gene rearrangements have been described, few of which have been extensively studied like *NPM1* (5q35)/*ALK* (2p23), which was the first identified *ALK* fusion to nucleophosmin (*NPM1*) in anaplastic large cell lymphoma [8] and *EML4* (2p21)/*ALK* (2p23) in non-small-cell lung cancer [9, 10]. *EML4-ALK* in non-small-cell lung cancer (NSCLC) serves as a diagnostic marker and therapeutic target. Advanced NSCLC associated with the *ALK* fusion oncogene is highly sensitive to ALK tyrosine kinase inhibitors (TKIs), such as ceritinib, alectinib, and brigatinib [11, 12].

Other described fusion genes are *RANBP2*(2q12*)/ALK(2p23)* associated with inflammatory myofibroblastic tumors; *ATIC(2q35)/ALK(2p23), TFG(3q12)/ALK(2p23),* and *MSN(Xq12)/ALK(2p23)* associated with anaplastic large cell lymphoma; *SQSTM1(5q35)/ALK(2p23)* associated with *ALK*-positive large B-cell lymphoma; *KIF5B(10p11)/ALK*(2p23) associated with NSCLC; *STRN(2p22)/ALK(2p23)* associated with DTC and breast cancer; *CLTC*(17q23)/*ALK(2p23)* and *TPM4(19p13)/ALK(2p23)* associated with anaplastic large cell lymphoma and inflammatory myofibroblastic tumors [13] (https://https://www.genecards.org/Search/Keyword?queryString=ALK%20EML); and Atlas of Genetics and Cytogenetics in Oncology and Haematology (atlasgeneticsoncology.org).

Our case's presentation was atypical for a well-differentiated FV-PTC in many aspects, including the rapid progression of clinical symptoms and signs, the extension beyond the thyroid and lymph nodes, and the persistence of high-volume local disease after thyroidectomy. Mutation analysis of the tumor tissue showed the presence of *EML4e13-ALKe20* fusion.

*EML4-ALK* fusion in thyroid cancer was first described in relation to exposure to ionizing radiation [14]. Hamatani et al. explored *EML4/ALK* and other *ALK* rearrangements in Atomic Bomb Survivors (ABS) of Hiroshima and Nagasaki who were diagnosed with PTC between 1956 and 1993. In the 25 ABS survivors with PTC with no detectable gene alteration in *BRAF*, *RAS*, *RET*, or *NTRK1*, *ALK* rearrangement was found in 10 out of 19 radiation-exposed PTCs (see Table 1). Five PTC cases with rearranged *ALK* were a fusion of *EML4* (exon 13) and *ALK* (exon 20), one case was a fusion of *EML4* (exon 20) and *ALK* (exon 20), and *ALK* partner genes in four cases have not been determined by the time of the publication and none in 6 patients with PTC without radiation exposure. Besides, solid/trabecular-like architecture in PTC was closely associated with *ALK* rearrangement in this cohort, being observed in 6 of 10 PTC cases with *ALK* rearrangements versus 2 of 15 cases with no *ALK* rearrangements [14].

Agrawal et al. in The Cancer Genome Atlas (TCGA) project performed a comprehensive multiplatform analysis of 496 PTCs. Fusions involving *ALK* presented in 4/484 (0.8%) tumors, including *EML4/ALK* [15].

Kelly et al. screened a total of 256 well-differentiated PTCs using whole-transcriptome RNA-sequencing analysis. The overall frequency of *ALK* fusion was four (1.6%) in 256 samples. *EML4-ALK* fusion was found in one tumor, and *ALK* fusions involving the striatin gene (*STRN-ALK*) were found in 3 tumors. In comparison, *STRN-ALK* was detected in three of 35 PDTCs (9%) and one of 24 ATCs (4%). All detected *ALK* fusions were *STRN-ALK*, and no additional cases of *EML4-ALK* were found [16].

Panebianco et al. showed in a series of 44 *ALK*-translocated thyroid neoplasms that *ALK* fusion-positive thyroid carcinomas are typically infiltrative PTCs with a common follicular growth pattern and may show tumor dedifferentiation, which is associated with increased mortality. The most common *ALK* fusion partners were *STRN* (*n* = 22) and *EML4* (*n* = 17). In 5 cases, novel *ALK* fusion partners were discovered. All 5 PDTCs carried the *STRN-ALK* fusion. Compared to *EML4-ALK, STRN-ALK* may be more common in PDTC, and ∼10% of *ALK* fusions involve rare gene partners [17]. In the same study, Panebianco et al. showed that all 19 resected thyroid nodules with *ALK* fusions identified preoperatively by fine-needle aspiration were malignant [17].

Chu et al. investigated the correlation between genotype and tumor behavior in 62 kinase fusion-positive thyroid carcinomas, including 57 papillary thyroid carcinomas (PTCs), two poorly differentiated thyroid carcinomas (PDTCs), two undifferentiated thyroid carcinomas (ATCs), and one primary secretory carcinoma (SC). *ALK* rearrangements were found in one PDTC and two PTCs. The two PTCs, despite having different fusion partners (*STRN* and *EML4*), were morphologically similar (with multinodular growth and predominantly follicular architecture) [6].

Point mutation of *ALK* gene has been reported by Murugan and Xing, who identified two novel point mutations in exon 23 of the *ALK* gene, *C3592T* and *G3602A*, in patients with anaplastic thyroid cancer with a prevalence of 11.11% but found no mutations in the matched normal tissues or in well-differentiated thyroid cancer [18].

## 4. Conclusion

In summary, we presented a patient with aggressive DTC with a less common gene fusion involving the *ALK* gene (*EML4e13-ALKe20* fusion). Identification of *ALK* rearrangement in thyroid tumor is of particular interest due to crizotinib's effectiveness and other specific ALK inhibitors that have been shown to induce a remarkable response in one case of aggressive PTC and ATC [18, 19]. Kelly et al. demonstrated that crizotinib effectively inhibits thyroid cell proliferation driven by *STRN-ALK*, raising the possibility that *ALK* may serve as a therapeutic target for thyroid cancer [16]. Godbert et al. demonstrated a remarkable response to crizotinib in women with anaplastic lymphoma kinase-rearranged anaplastic thyroid carcinoma [20].

Precision medicine is an emerging approach for disease management that considers individual variability in presentation, progression, and response to treatment. Genetic mutation analysis and molecular markers are the cornerstones of precision medicine in cancer treatment, as many of these mutations are considered therapeutic targets for modern cancer medications.

The selective use of molecular diagnostics can help with DTC management, especially in patients with atypical presentations or more aggressive clinical courses.

## Figures and Tables

**Table 1:** Prevalence of *EML4/ALK* fusion.

Author (reference)	Year	Country	Study population	Sample size	Number of persons with kinase gene rearrangement (%)	Patients with *EML4/ALK* (number)	Findings	Follow-up (months)
Chu et al. [6]	2020	USA	PTC, PDTC, ANC	395	62 (16%)	1	Association seen between kinase gene fusion and clinical aggressiveness	6–480
Hamatani et al. [14]	2012	Japan	Atomic bomb survivors with PTC	105	10 (10%)	6	ALK fusion associated with radiation	NA
Agrawal et al. [15]	2014	USA	Patients with PTC	496	74 (15%)	4	>96% of PTCs have driver oncogenic mutation	NA
Kelly et al. [16]	2014	USA	PTC	256	4 (2%)	1	Compared to EML4-ALK, STRN-ALK is more common in PDTC	NA
Panebianco et al. [17]	2019	USA	PTC and PDTC	44	44 (100%)	17	Compared to EML4-ALK, STRN-ALK is more common in PDTC	2–108 months

PTC = papillary thyroid cancer; ATC = anaplastic thyroid cancer; PDTC = poorly differentiated thyroid cancer.

## Data Availability

All data generated or analyzed during this study are included in this published article or in the data repositories listed in references.
